# ‘Because the baby asks for it’: a mixed-methods study on local perceptions toward nutrition during pregnancy among marginalised migrant women along the Myanmar–Thailand border

**DOI:** 10.1080/16549716.2018.1473104

**Published:** 2018-05-22

**Authors:** Ahmar H. Hashmi, Moo Kho Paw, Suphak Nosten, Mu Chae Darakamon, Mary Ellen Gilder, Prakaykaew Charunwatthana, Verena I Carrara, Kremlin Wickramasinghe, Chaisiri Angkurawaranon, Emma Plugge, Rose McGready

**Affiliations:** a Shoklo Malaria Research Unit, Mahidol-Oxford Tropical Medicine Research Unit, Mahidol University, Mae Sot, Thailand; b Department of Family Medicine, Faculty of Medicine, Chiang Mai University, Chiang Mai, Thailand; c Mahidol-Oxford Tropical Medicine Research Unit, Mahidol University, Bangkok, Thailand; d WHO European Office for the Prevention and Control of Non-Communicable Disease, Moscow, Russia; e Centre for Tropical Medicine and Global Health, Nuffield Department of Medicine, University of Oxford, Oxford, UK

**Keywords:** Nutrition in pregnancy, dietary preferences, eating behaviours, nutrition awareness

## Abstract

**Background**: Under- and over-nutrition during pregnancy are known risk factors for pregnancy complications and adverse pregnancy and infant outcomes. Understanding perceptions around nutrition in pregnancy can create culturally appropriate interventions for improved health outcomes.

**Objective**: A mixed-methods study was performed to explore local perceptions and practices of diet and physical activity in pregnancy in a marginalised population along the Myanmar–Thailand border.

**Methods**: From April to July 2017, a cross-sectional survey and focus group discussions were conducted with pregnant women reporting to antenatal care; in-depth interviews were conducted with senior midwives at participating organisations along the Myanmar–Thailand border.

**Results**: A total of 388 pregnant women were interviewed at two clinic sites along the Myanmar–Thailand border. A high proportion of women had limited knowledge of and poor dietary practices. Consuming a sweetened drink in the last 24 hours as well as being a non-teenage, multigravida woman was significantly associated with high body mass index (BMI) compared to normal BMI. Qualitative analysis combined focus group discussions (n = 66) and in-depth interviews (n = 4) summarising emergent themes: common foods eaten or avoided and rationale; benefits of nutrition; perceptions of overweight and weight gain during pregnancy; barriers to a healthy diet; and sources of diet information.

**Conclusions**: There is limited awareness about healthy diets and lifestyle in these marginalised, migrant communities along the Myanmar–Thailand border. This study suggests that simple, culturally appropriate messaging should be provided to women and communities with low health literacy to generate awareness about healthy lifestyles and their effects on pregnancy outcomes as an important element of a broader strategy to address maternal nutrition in this population. However, more studies to determine the effectiveness of a broad range of interventions in low- and middle-income countries (LMIC) are needed, especially in marginalised migrant populations.

## Background

The growing double burden of under- and over-nutrition in pregnant women in low- and middle-income countries (LMIC) has a direct impact on maternal, infant and child health [1–4]. Infant health and child development closely relate to maternal nutrition, with effects extending into adolescence and adulthood [5–7]. These impacts have important societal and economic implications, resulting in reduced human capital – a cycle of malnutrition with intergenerational effects most felt in (LMIC) [8].

Among LMIC, Asian populations are disproportionally affected by the nutrition transition due to increased risk of morbidity from diet-related diseases of over-nutrition at lower BMI than non-Asians. These diseases of overweight coexist with persistently high rates of under-nutrition in Asia [9–13]. As the global poor bear the greatest burden of risk due to the nutrition transition [14], migrant populations within Asia require special attention. In 2015, migrants numbered 244 million globally, after rapid increases in the preceding 15 years [15,16]. Along the Myanmar–Thailand border a large workforce of low-skilled labourers from Myanmar supports Thailand’s burgeoning professional and service industries [17–20]. In 2016, Myanmar made up 80% of all documented migrants in Thailand (1.43 million), with an additional estimated 1–2 million undocumented migrants in Thailand from Myanmar, Cambodia and Lao PDR [21]. Migrants face significant constraints on their social well-being with particular concern for pregnant women who have limited options for appropriate care during pregnancy [22–25] and restricted access to healthy foods. Recent analyses of body mass index (BMI) and gestational weight gain (GWG) over the past 30 years among migrant and refugee pregnant women along the Myanmar–Thailand border mirror global trends in persistent under-nutrition with a rapid rise in overweight and obesity [26].

Although nutrition is a key modifiable risk factor in pregnancy, little is known about the diet of pregnant women in LMIC and particularly pregnant migrants. Despite the 2009 Institute of Medicine (USA) guidelines on GWG, health workers in high-income countries (HIC) report difficulties in antenatal nutrition counselling [27–29]. These challenges are exacerbated in LMIC settings where poverty and poorly resourced health systems can perpetuate a lack of awareness about the importance of nutrition and physical activity amongst health workers and local communities [30–33]. Although rare in the literature, a focus on marginalised populations such as migrants and refugees from a region of economic inequality and armed conflict can provide insights into social, ethnic and cultural influences on maternal nutrition [34,35]. To inform the development of targeted interventions around maternal nutrition, a mixed-methods study was conducted to examine common perceptions and practices of diet and physical activity among pregnant women in a migrant population along the Myanmar–Thailand border.

## Methods

### Setting

**Figure 1.**
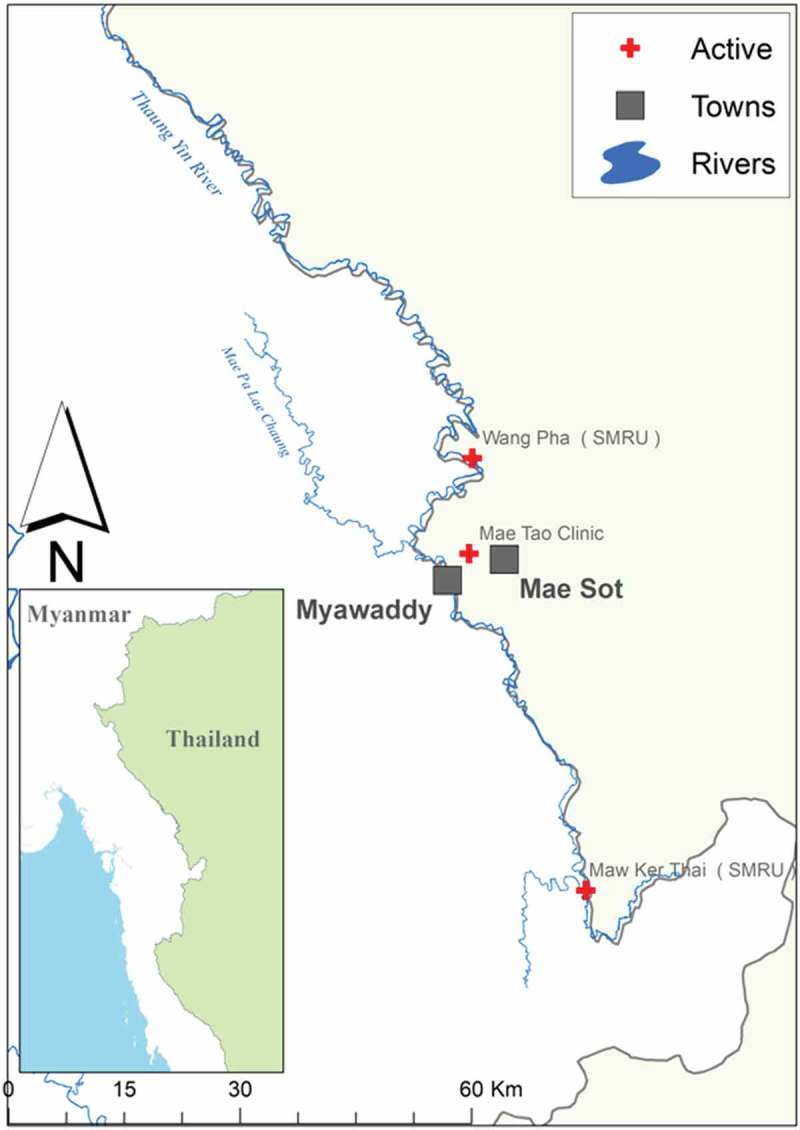
Map of ANC sites included in study. Credit: Myo Chit Min.

A cross-sectional survey and focus group discussions (FGD) with pregnant women were conducted at two antenatal care (ANC) clinics run by the Shoklo Malaria Research Unit (SMRU) in Maw Ker Thai (MKT) and Wang Pha (WPA), Thailand. SMRU has provided medical and obstetric services for migrants since 1998 in MKT and 2004 in WPA. In-depth interviews (IDI) were conducted with midwives from SMRU and Mae Tao Clinic (MTC). MTC has been providing care for migrant communities in urban border areas since 1989 (Figure 1). This post-conflict region has emerged from more than five decades of civil unrest due to differences with the central government of Myanmar. Education systems have suffered and economic inequalities have prompted many to seek part-time residence along the border, where health services remain limited in rural and remote areas. SMRU and MTC have provided free maternal and child health services for decades to fill a gap otherwise unanswered by governments on either side of the border [17–20].

In the SMRU and MTC catchment areas, migrants residing on both sides of the border frequently move to and from Myanmar but the total population, estimated at 200,000, is relatively stable [19,36,37]. In 2016, SMRU performed over 16,000 ANC consultations and over 1600 deliveries. MTC provided over 10,000 ANC consultations and 2500 deliveries in the same year [38]. Pregnant women receiving care reflect the migrant populations along the border, who are predominantly Burman or Karen; commonly speak Burmese, and Sgaw and Pwo Karen; and represent Buddhists, Christians and Muslims [37]. Average socio-economic status of women from SMRU WPA ANC is higher than MKT, but both communities have low literacy rates at around 45% [39]. Women presenting to SMRU ANC have a median of 11 visits prior to delivery [40].

### Study design

A convergent parallel mixed-methods design was employed with quantitative (cross-sectional survey), and qualitative (FGD, IDI) components exploring pregnant women’s perceptions and behaviours regarding nutrition in pregnancy. The mixed-methods approach was chosen to enhance findings in quantifying behaviours and perceptions around nutrition and exercise with qualitative components exploring the rationale for these behaviours and perceptions. Prior to finalisation, cross-sectional survey and FGD and IDI guides were translated into Sgaw Karen and Burmese, back-translated to identify and correct errors in translation or meaning, and piloted with 20 pregnant women or SMRU staff.

#### Cross-sectional survey

All pregnant women presenting to ANC were approached individually and informed about the study. The aim was to conduct 200 surveys from each SMRU clinic as this approached the total number of monthly ANC consultations. Women presenting for delivery or pregnancy complications were excluded. Four study staff were trained at each site to administer the questionnaire in the patients’ preferred languages. Informal investigator observations informed the survey, which consisted of 12 questions evaluating women’s knowledge of appropriate GWG, their pre-pregnancy weight, if they returned to pre-pregnancy weight after prior delivery (for multigravida) and if they felt they knew what constitutes a healthy diet. Women were also asked if they perceived themselves to be active and about ‘healthy behaviours’ such as: ever having exercised and, if so, how often; most recent consumption of a snack or a sweetened beverage. Snacks (small amounts of food between larger meals) were recorded by interviewers and categorised as healthy or unhealthy; photographs of locally available sweetened beverages were shown to women to easily identify what they consumed.

#### Focus group discussions

**Figure 2.**
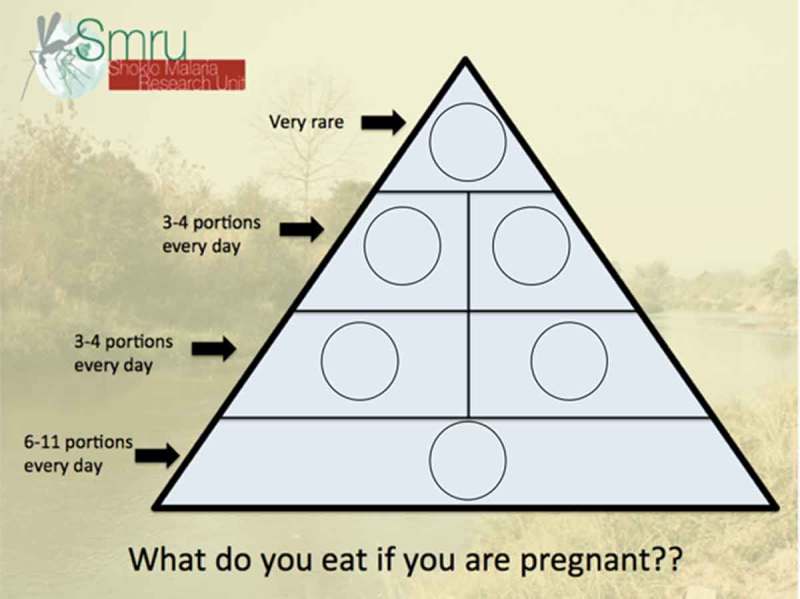
Food pyramid puzzle used in FGD with pregnant women.

The FGD facilitator was trained and experienced in leading FGD, and fluent in Burmese, and Sgaw and Pwo Karen. When possible, an additional facilitator fluent in the three languages also helped with FGD and assisted in note-taking by the primary author. FGD were conducted in private counselling rooms, once participants consented to being audio-recorded. FGD began with women prompted to solve a food pyramid puzzle with photos of food groups according to their usual dietary preferences (Figure 2), followed by questions and probes as outlined in Table 1. Pregnant women presenting to ANC were approached, provided with information and, if expressing interest, these women were grouped according to age, parity, ethnicity and language. If groups of four to eight participants were possible among women presenting to ANC on a given day, these women provided consent and FGD were then conducted.

**Table 1.** Focus group discussion guide used with pregnant women at SMRU ANC in Maw Ker Thai and Wang Pha.

Question	Probes
Food pyramid puzzle	What? Why? Do you eat differently? Is this good or bad?
Do you eat like this?	What? Why? Do you eat differently?
Did you eat like this today?	Yesterday? What are the foods you crave? Is this good or bad?
How many meals do you eat in a day?	How much do you eat at each meal? Do you eat snacks? What snacks?
What about other pregnant women: what do they eat?	Do you get advice on what to eat? What advice? Are there any special foods to avoid?
What do you think about gaining weight during pregnancy?	Can you eat too much during pregnancy? What can happen to you? Your baby?
Look at the puzzle: is it possible to eat like this every day?	Why/Why not? What are other reasons you can/cannot eat like this? Do you have support at home? How does it affect your diet?

#### In-depth interviews

SMRU and MTC senior midwives (≥ 8 years of experience) were invited to participate in IDI to discuss changes seen in pregnant women nutrition over their careers. Questions explored midwives’ perceptions of nutrition and dietary habits of pregnant women; and changes in pregnant women nutritional status, GWG and access to healthy foods over time. Ideas about appropriate counselling measures or interventions for nutrition in pregnancy were also discussed. All midwives were provided with information on the study prior to consenting to participate, agreed to have IDI recorded and were interviewed in a private room at SMRU or MTC clinics.

#### Anthropometric measurements

Trained midwives collected maternal weight and height data at the first ANC visit in the first trimester and this was used to estimate pre-pregnancy BMI, if available [41,42]. A mechanical scale measured weight accurate to 0.5 kg. A stadiometer measured height with precision up to 1 mm. World Health Organization (WHO) standards for Asian populations were used to determine low (< 18.5 kg/m^2^), normal (18.5–23 kg/m^2^) and high (≥ 23 kg/m^2^) BMI.

### Statistical analysis

Cross-sectional survey data were compared using Student’s t-test for continuous, normally distributed variables, and the Chi-squared test for categorical variables. Bonferroni correction was applied to baseline characteristics with multiple comparisons (Table 2).

**Table 2.** Demographic characteristics of all women participating in the cross-sectional survey and by BMI category (MKT = Maw Ker Thai ANC; WPA = Wang Pha ANC).

		All women	BMI category		
		n = 388	Low (< 18.5 mg/kg^2^) n = 54	Normal (18.5–23 mg/kg^2^) n = 231	High (> 23 mg/kg^2^) n = 103
Site	MKT	203 (52.3)	23 (42.6)_a_	126 (54.5)_a_	54 (52.4)_a_
	WPA	185 (47.7)	31 (57.4)_a_	105 (45.5)_a_	49 (47.6)_a_
Ethnicity^a^	Burmese	161 (41.5)	33 (61.1)_a_	91 (39.4)_b_	37 (35.9)_b_
	Karen	206 (53.1)	17 (31.5)_a_	130 (56.3)_b_	59 (57.3)_b_
	Other	21 (5.4)	4 (7.4)_a_	10 (4.3)_a_	7 (6.8)_a_
Age mean ± SD [min-max] (yrs)		28 ± 7 [16–45]	25 ± 6 [16–41]_a_	25 ± 6 [16–43]_a_	28 ± 7 [16–45]_b_
	< 20 years	72 (18.6)	15 (27.8)_a_	47 (20.3)_a,b_	10 (9.7)_b_
	≥ 20 years	316 (81.4)	39 (72.2)_a_	79.7 (184)_a,b_	93 (90.3)_b_
Gravidity	Primigravida	164 (42.3)	31 (57.4)_a_	106 (45.9)_a_	27 (26.2)_b_
	Multigravida	224 (57.7)	23 (42.6)_a_	125 (54.1)_a_	76 (73.8)_b_
Work status^b^	Unemployed	192 (49.5)	33 (61.1)_a_	112 (48.5)_a_	47 (45.6)_a_
	Employed	196 (50.5)	21 (38.9)_a_	119 (51.5)_a_	56 (54.1)_a_
First ANC visit	Trimester 1	188 (48.5)	36 (66.7)_a_	115 (49.8)_a,b_	37 (35.9)_b_
	Trimester 2	175 (45.1)	18 (33.3)_a_	106 (45.9)_a_	51 (49.5)_a_
	Trimester 3	25 (6.4)	0 (0.0)	10 (4.3)_a_	15 (14.6)_b_

No missing data; data presented as number (percentage) unless stated otherwise

^a^‘Other’ ethnicity includes Hmong, Ka May, Mon, Pa Oh, Rakhine, Kachin, Burmese-Muslim.

^b^Employed=salaried work; Unemployed=house and family work, i.e., not salaried. [hard return]

_a,b_ Values in the same row not sharing the same subscript are significantly different at p < 0.05 in the two-sided test of equality for column proportions or for the mean, using the Bonferroni correction for multiple comparisons.

No subscript indicates the value was not included in the comparison.

Cross-sectional survey responses were summarised for all women and for those who first presented to antenatal care in the first trimester (Table 3). BMI taken after the first trimester is no longer a proxy for pre-pregnancy BMI; therefore, only women enrolled in the first trimester were included (Table 3). Logistic regression was used to examine associations between BMI (low vs normal and high vs normal) and cross-sectional survey responses. Both models were adjusted for ethnicity (Burman, Karen or other). Age < 20 years (teenage) and gravidity demonstrated collinearity and a combination variable was used in the model: teenage and primigravida, teenage and/or primigravida, and neither teenage nor primigravida. Covariates that were significant at p < 0.10 were included in a multivariate logistic regression model where, using a stepwise approach, only those variables that were significant at p < 0.05 were retained in the final model and considered independent risk factors. Factors significantly associated with high BMI in the first trimester were reported as adjusted odds ratios (AOR) with 95% confidence intervals (95% CI). Data from the cross-sectional survey were analyzed using Stata v14.0 (StataCorp, College Station, Texas, USA) and SPSS v15.0 (IBM, Armonk, New York, USA).

**Table 3.** Responses to selected cross-sectional questions on perceptions and behaviours regarding nutrition in pregnancy at SMRU ANC.

							p-value		
		All women	First trimester	BMI category			High vs Normal		Low vs Normal
		N = 388	N = 188	Low n = 36	Normal n = 115	High n = 37	Univariate	Adjusted OR^c^	Univariate
Knows appropriate GWG	Yes	57 (14.7)	28 (14.9)	4 (11.1)	18 (15.7)	6 (16.2)	0.935	Excluded	0.500
	No	331 (85.3)	160 (85.1)	32 (88.9)	97 (84.3)	31 (83.8)			
Knows pre-pregnancy weight	Yes	203 (52.3)	103 (54.8)	16 (44.4)	66 (57.4)	21 (56.8)	0.946	Excluded	0.174
	No	185 (47.7)	85 (45.2)	20 (55.6)	49 (42.6)	16 (43.2)			
Return to pre-pregnancy weight	Yes	149/224 (66.5)	65/102 (63.7)	9 (60.0)	45 (77.6)	11 (37.9)	<0.001	Excluded	0.166
	No	75/224 (33.5)	37/102 (36.3)	6 (40.0)	13 (22.4)	18 (61.2)			
Perceived active	Yes	299 (77.1)	143 (76.1)	25 (69.4)	89 (77.4)	29 (78.4)	0.900	Excluded	0.333
	No	89 (22.9)	45 (23.9)	11 (30.6)	26 (22.6)	8 (21.6)			
Ever exercised	Yes	113 (29.1)	58 (30.9)	9 (25.0)	38 (33.0)	11 (29.7)	0.708	Excluded	0.363
	No	275 (70.9)	130 (69.1)	27 (75.0)	77 (67.0)	26 (70.3)			
Knows a healthy diet	Yes	219 (56.4)	107 (56.9)	16 (44.4)	70 (60.9)	21 (56.8)	0.657	Excluded	0.082
	No	169 (43.6)	81 (43.1)	20 (55.6)	45 (39.1)	16 (43.2)			
Ate a snack in the last 24 h	Yes	301 (77.6)	144 (76.6)	30 (83.3)	89 (77.4)	25 (67.6)	0.230	Not-significant	0.446
	No	87 (22.4)	44 (23.4)	6 (16.7)	26 (22.6)	12 (32.4)			
Regularly eats a healthy snack^a^	Yes	141 (36.7)	70 (37.2)	13 (36.1)	40 (34.8)	17 (45.9)	0.245	Not significant	0.884
	No	243 (63.3)	118 (62.8)	23 (63.9)	75 (65.2)	20 (54.1)			
Consumed sweet drink	No	26 (6.7)	8 (4.3)	1 (2.8)	3 (2.6)	4 (10.8)	0.010	Referent	0.897
	≤24h	186 (47.9)	91 (48.4)	15 (41.7)	53 (46.1)	23 (62.2)		2.86 (1.17–7.01), p = 0.022	
	>24h	176 (45.4)	89 (47.3)	20 (55.6)	59 (51.3)	10 (27.0)		0.44 (0.08–2.40), p = 0.340	
Teenager (T) and/or Primigravida (P)	T + P+	72 (18.6)	38 (20.2)	11 (30.6)	24 (20.9)	3 (8.1)	0.008^b^	Referent	0.315
	T+ or P+	100 (25.8)	52 (27.7)	12 (33.3)	34 (29.6)	6 (16.2)		3.46 (0.87–13.65), p = 0.076	
	T- P-	216 (55.7)	98 (52.1)	13 (36.1)	57 (49.6)	28 (75.7)		2.89 (1.02–8.16), p = 0.046	

Missing data: Regularly eats a healthy snack n = 4 missing (reports consuming a snack but actual snack food not reported).

Data presented as number (percentage) unless stated otherwise.

Abbreviations: BMI category = low < 18.5, normal 18.5–23.0, and high ≥ 23.0 kg/m^2^; GWG = gestational weight gain; T + P+ = teenager AND primigravida; T+ or P+ = teenager OR primigravida; T- P- = not a teenager AND not primigravida.

^a^Regularly eats a healthy snack or does not snack.

^b^p-value for linear trend.

^c^Adjusted for ethnicity.

The recordings of FGD and IDI were transcribed from Karen and directly to English by AHH working with translators fluent in Burmese and Sgaw and Pwo Karen. Line-by-line inductive coding was initially performed on full transcripts of FGD and IDI by AHH. Any outstanding questions, clarifications and confirmation of findings from the initial transcription and coding phases were discussed with AHH, MKP, SP and MCD until consensus was reached. Thematic analysis based on initial coding was used to identify emergent themes and triangulate the information collected [43,44] facilitated by NVivo for Mac v11.4.0 (QSR International, Melbourne, Australia). Finally, queries were performed on textual data to identify variations across pregnant women according to parity, ethnicity and site as well as for midwives. This inductive approach allowed for establishing links between research objectives and findings from raw data and developed deeper understanding of the underlying experiences and processes evident in emergent themes.

## Results

### Cross-sectional survey

A total of 388 pregnant women participated in cross-sectional surveys in MKT (n = 203) and WPA (n = 185; Table 2). At each site, most pregnant women meeting inclusion criteria were included: 72.2% (203/281) in MKT and 59.9% (185/309) at WPA. Significant differences between BMI groups were observed in ethnicity, gravidity, age (and age < 20 years) and trimester of first ANC attendance (Table 2). Of the pregnant women who reported employment the majority 85.2% (167/196) were in unskilled jobs including daily manual labour, factory work and agricultural work.

Overall, pregnant women had limited knowledge and poor dietary practices (Table 3). A high proportion of women did not know what constitutes a healthy diet (43.6%; 169/388) and were unsure of an appropriate GWG (85.3%; 331/388), and nearly half (47.7%; 185/388) of the women did not know their pre-pregnancy weight. Three-quarters (77.1%; 299/388) perceived they were active but less than one-third (29.1%, 113/388) reported ever exercising. Most women (63.3%; 243/384) reported regularly eating unhealthy snacks, with almost half (47.9%; 186/388) of the women having consumed a sweetened beverage in the 24 hours prior to being surveyed. Of the 14% (55/388) of women who provided an estimate of appropriate GWG, the median weight nominated was 6 kg (range 0–20 kg).

The proportion of first antenatal care visits was highest for the first trimester than in the second or third trimesters (Table 2). In the sub-cohort of women with BMI measured in the first trimester, the proportion of women who had consumed sweetened beverages in the previous 24 hours was highest in the high BMI group (Table 3). Variables excluded in regression analysis were knowledge of GWG, pre-pregnancy weight, return to pre-pregnancy weight, women’s perception of activity, ever exercised, or knowledge of a healthy diet. Consuming a sweetened drink and age (teenager) with gravidity were initially included in the regression model with covariates for women consuming a snack in the prior 24 hours and eating unhealthy snacks. Consuming a sweetened drink in the last 24 hours as well as being a non-teenage, multigravid woman was significantly and independently associated with high BMI compared to normal BMI, and there was no association with consuming a snack in the prior 24 hours and/or eating unhealthy snacks after adjustment (Table 3). The proportion of women who did not know what constitutes a healthy diet was highest in the low BMI group but this was not statistically significant (Table 3). Amongst multigravida, the proportion of women self-reporting not returning to their pre-pregnancy weight was higher among women with high BMI than with normal BMI (62.1% vs. 22.4%, AOR 5.66, 95% CI 2.14–14.96; p = 0.001) (Table 3).

### Qualitative findings

A total of 11 FGD of four to eight participants each were conducted with a total of 66 women; 38 from a total of 7 FGD from MKT and 28 women from a total of 4 FGD from WPA ranging in age from 18 to 39 and 18 to 40 years, respectively. Two FGD were conducted with Burman primigravida; and three FGD conducted with Karen primigravida, Karen multigravida and Burman multigravida each. IDI were conducted with four midwives; two from SMRU and two from MTC.

#### Foods eaten

Staple foods for pregnant women across all groups included rice, vegetables, fruits and milk. Common sources of protein included fish, chicken and eggs, but were often limited in pregnant women’s diets. Karen and primigravida women were more likely to mention protein sources in their diet. Women attributed limited intake of protein to preference, noting that the smell of raw or cooked meats was offensive and nauseating. Most milk consumed by women is processed and high in sugar and/or saturated fats. Burman women made more mention of curries in their diets, which often were served with rice. Oil, and in particular palm oil, was often included in the diet of women from WPA. Although many women desired soybean and other vegetable oils as they were considered healthier, palm oil was more often used due to cost and availability.

When asked about snacks, women mentioned regularly eating small amounts of food between meals, but also included beverages such as milk and other sweetened beverages (e.g. Sprite). Often women consumed cheap snacks purchased at local shops that are deep-fried, artificially sweetened, industrially or locally processed and/or high in sugar or salt content. Burman women and women from MKT spent the greatest amount of time discussing snacks, citing their inexpensiveness, ability to satiate a craving or hunger quickly and as an option when appetite is low as in the earlier stages of pregnancy:

Yes, I eat one time a day, but I cannot eat much so I eat only a little. I eat snacks meant for children – 1 packet for 5 baht [USD $0.15] – and cake [processed, sweetened].

I eat a little bit of rice, but more fruits. (Primigravida, Karen, MKT)

I eat rice only a little bit. Normally I drink milk and eat snacks. (Burman, Multigravida, MKT)

#### Benefits of nutrition

As a rationale for their diet, women commonly noted that they would ‘think’ that these were the appropriate foods. Many reported indulging cravings, which were often attributed to the developing foetus (‘the baby asks for it’). There was a commonly held belief that during pregnancy a woman was free to eat what she pleased as her appetite allowed:

‘During pregnancy, we can eat everything, because the baby asks for it. If I see it, I will buy and eat.’ (Primigravida, Karen MKT)

Few women mentioned direct benefits of maternal nutrition, but many seemed to understand that diet could have benefits for maternal and foetal health. Many made simplified assertions that women should eat certain foods to ‘give the baby strength’, ‘to give the mother strength’ or to have ‘good energy’. A minority of women mentioned that meats, vegetables and fruits were important for development of the ‘baby brain’ and for the ‘baby to grow tall’.

#### Foods avoided

The main foods avoided were chilli, coconut water, dogfruit (*Archidendron pauciflorum*), ‘hot foods’, *daw kha tha* (*Oroxylum inidicum*) and raw foods. Chilli was universally avoided in its raw form to avoid baby discomfort (e.g. ‘burn the baby’). This did not preclude women from eating spicy foods, particularly if they were prepared with dried chillies (e.g. *nya oo htee* [Karen] or *nga pi* [Burmese], a fish paste prepared with fish sauce, shrimp paste and dried chillies). Coconut water, especially in early pregnancy, was commonly associated with miscarriage and a ‘difficult delivery’. Dogfruit was also mentioned as a common food to avoid in all groups. Its strong smell and bitter taste could harm the baby or cause a difficult delivery. Food items categorised as ‘hot foods’ to be avoided to minimise mother and baby discomfort included chillies (raw), spicy foods, ginger, coconut water and betel nut.

A legume found along the border region, *Oroxylum indicum* (Karen: *daw ka tha*), was believed by Karen women to cause miscarriage. One mother asserted that even sitting beneath a *daw ka tha* tree may induce miscarriage.

Raw foods, such as raw vegetables (including eggplant, commonly eaten raw along the border) as well as meats, were thought to cause disease (e.g. ‘worm infections’) in the mother or infant.

Another category mentioned included ‘sticky foods’, such as sticky rice and ripe bananas, as potentially causing a ‘sticky placenta’ that would make delivery difficult. These foods were also thought to be related to the amount of vernix on the delivered infant.

#### Overweight or obesity and weight gain during pregnancy

Most women did not know of an effect of excessive GWG on maternal or foetal/infant health. Women were evenly divided between viewing overweight or ‘fat’ as beneficial or harmful in pregnancy:

If you eat more then you will put on weight. The baby will become strong and you will become fat. For people who don’t want to eat or don’t like eating because of a lack of appetite – then in such cases you cannot add weight. (Primigravida, Karen, MKT)

However, some women feared that overweight or obesity risked a large infant and difficult delivery. Many could state direct risks to maternal health: pre-eclampsia (‘high blood pressure’), diabetes (‘sweet blood’ or ‘sweet urine’) and atherosclerosis (‘veins can become clogged’). Even upon probing, however, many were unaware of any relation of overweight or excessive GWG to infant health.

Midwives knew the ill effects of overweight and poor and/or excessive GWG to both mother and foetus. Midwives noted that overweight was of increasing concern compared to earlier years working in the region, when the primary concern was under-nutrition:

The mother has become too fat so it can be a problem when she comes to delivery. If there is too much fat it can lead to bad health. Over sweetness, diabetes. It can be that the fat closes the blood vessels. It can also cause joint pains. They can have sweet blood [diabetes] or high blood pressures. (Midwife, SMRU)

#### Social considerations and sources of information

Social factors impacting nutrition for pregnant women related to financial, food access and family considerations. Women most readily mentioned financial limitations, unprompted, especially among Karen women and women from MKT. Expense limited dietary choices such as meat, often consumed once or twice a week – corroborated by midwives. Often, finances fluctuated daily and women spent money on foods as it became available. Women often borrowed money for food from close relatives. Financial and logistic constraints commonly hampered access to foods, especially for those who lived far from urban areas. Family considerations were important: where household chores were shared with mothers, husbands, aunts or other family members, greater time was available for preparation of regular meals. Other members of the family may be responsible for the purchase of foods (e.g. the husband) or cooking (e.g. mothers or sisters). Some women, unable to work or prohibited from work, were dependent on other family members for their meals. Women and midwives mentioned gathering wild vegetables near one’s house to address financial constraints:

All of us who come here[,] none of us live in the towns, all of us live in different places and villages – country people. The foods that we are talking about … if people mentioned it, we can buy it to eat. But if they were not mentioned, then we cannot buy it. It doesn’t mean that we can afford everyday because we cannot. Where I live is now considered close to Koh Koh [near the SMRU WPA clinic, on the Myanmar side] but you have to take a motorcycle to get there and it costs 100 baht [USD $3.17]. So in order to eat all these things we have to travel some distance. For those who live far, far away it is out of the question. (Multigravida, Karen, WPA)

Midwives felt that better formal education and improved counselling by health workers were responsible for women having healthier diets than in the past:

‘Before, women were afraid of eating, but we have been teaching the women and now they have greater health awareness, fewer problems and the women are eating better’ (Midwife, MTC).

Commonly cited sources for information on foods to eat or avoid and nutritional benefits were elders within the community and family members. However, women frequently mentioned that their thoughts concerning appropriate foods for pregnant women came from no source at all (i.e. ‘I think by myself’), or that they ‘never heard’ about appropriate nutrition in pregnancy. Health professionals as a source of information were predominantly mentioned by multiparous women, likely reflecting more frequent contact with the health system:

For us, there are no specific things to eat, it is only if I think the food is nutritious for the baby, then I will try to eat it. (Multigravida, Burman, WPA)

For me, I have learned this information … whenever I heard it from the medic or health workers. When they talk about it, I try to pay attention and remember. (Multigravida, Burman, WPA)

Midwives and pregnant women agreed that information reflective of older traditions and beliefs circulates in communities, but that younger women often disregard such advice. Women appeared to consider information provided by community members to be less trustworthy than that of health professionals:

‘But [beliefs of the elders] are from before, and now we see that pregnant women eat everything, whatever they want to eat. These are the changes; they eat everything’ (Midwife, MTC).

#### Counselling considerations

The food pyramid game (Figure 2) helped assess what women ate and explored women’s knowledge and awareness, yielding insights about counselling. The instructions for the game were repeated up to three times in each FGD, with the pyramid described as relative frequency of food groups eaten, rather than portions of each food group per day. This is likely a reflection of low health literacy and numeracy among this population, as well as cultural differences in measurement or awareness of amounts of foods included in one’s diet. The vocabulary of pregnant women did not appear to allow for certain concepts; for example, photos depicting protein were often referred to as ‘meat’, although these photos included lentils and eggs.

When asked about how to improve pregnant women nutrition, midwives suggested clinic-based counselling, having noticed benefits of individual counselling using posters, flip charts and handouts. One midwife mentioned the potential benefits of group counselling:

‘Sometimes, we counsel the breastfeeding women in a group. We can do this type of counseling. We can explain, “How should women eat?” “What to eat?” ’ (Midwife, SMRU).

Two midwives shared reservations about their expertise on dietary recommendations for pregnant women. Two midwives noted limitations in time available for clinic-based counselling or funds to provide dietary supplements. Midwives were quick to suggest that women could eat nutritious foods, but that many lacked awareness, stating that most pregnant women would try to adhere to midwives’ recommendations, as financial constraints allowed:

We encourage them that if they have money they should buy the healthy foods, but if you cannot afford, you can try to pick vegetables in the forest that grow for free. There are plenty. We do counselling like this. (Midwife, MTC)

## Discussion

This study examines perceptions and behaviours related to nutrition in pregnancy in a marginalised community along the Myanmar–Thailand border. Both the quantitative and qualitative findings suggest a dearth of awareness about nutrition. Among women with BMI available from the first trimester, the survey showed that being overweight was associated with consumption of sweetened beverages, being a non-teenage multigravida, and not returning to pre-pregnancy weight (among multigravida). Many women expressed a limited understanding of the link between maternal nutrition and foetal development. Although better informed, midwives lacked confidence in counselling pregnant women on appropriate nutrition. Considering the limitations of counselling by health workers in HIC, a feeling of inadequacy among local midwives is not surprising in this low-resource context with a rapidly changing burden of diet-related disease [27,28,32].

Information on healthy and unhealthy foods was variable. Women mentioned social dietary taboos, which provide a cultural ‘awareness’ regarding specific ‘unhealthy’ local foods, intended to protect mother and child [45,46]. As in other Asian populations [47], meat as a protein source was limited due to pregnant women’s preference and its cost, with health benefits largely unknown. Likewise, Karen and Burman women expressed concern over ‘hot’ and ‘cool’ foods – likely alluding to a perceived internal bodily balance [46]. However, women often disregard dietary taboos [46]. Perceptions of healthy and unhealthy foods change over time, as described elsewhere in South Asia; women now may consider eating fruits and vegetables to be healthy, whereas previously they avoided these foods as a means of ‘eating down’ [48–50].

The wider public health issue along the border is the ease of access to cheap, calorically dense, processed foods with adverse nutritional effects that exacerbate a lack of awareness and proclivity to indulge cravings [13,51–53]. This becomes increasingly important in a context where women are restricted financially and geographically in accessing healthier foods. Pregnant women and midwives posited that women would make improved dietary choices, within their social constraints, if they were provided with appropriate information regarding nutrition. However, the reach of clinic-based care during pregnancy is far from comprehensive in this border region, with limitations in clinic-based programming for maternal nutrition [24,54,55]. Awareness-raising at a community level should be considered to reach younger women whose diets may be tied to the choices of older members of the household.

Our findings suggest that simple, culturally appropriate counselling measures should be provided for women with low health literacy in LMIC settings [30,31,34,39]. The puzzle engaged women in discussion and identified gaps in knowledge about nutrition. Pictures are key in settings where numeracy and measurements for food frequency among pregnant women may be limited. Clear, broad prescriptions about foods high in sugar, salt or fats can help women make appropriate dietary choices when faced with easy access to processed foods. Such instructive measures lend themselves to group counselling in clinics or women’s groups at a community level [56]. This requires developing the capacity of local health workers in effective counselling and should make use of older women equipped with appropriate information when possible [57]. Continued research on pregnant women nutrition along the border could produce easy-to-understand, culturally and contextually specific information materials to counsel women presenting to ANC about healthy GWG [26,27,58]. However, recent analyses suggest that such lifestyle measures are of modest benefit during pregnancy [34,59,60] and change will likely require policy interventions to improve access to healthy foods along the border to influence pre-conception maternal nutrition [61]. Similar upstream interventions should focus on accessing and educating women prior to conception, as pre-conception care is exceedingly rare in these communities [55]. Such far-reaching public health interventions have the potential to break the intergenerational cycle of malnutrition and poverty.

The mixed-methods study design allowed quantitative measures of behaviours and potential impact on BMI to be further explained by qualitative methods [62]. All methods allowed for triangulation of the main study findings. Study limitations include limited generalisability due to geographic and cultural specificity [32], and lack of external validation of the cross-sectional survey. Regression analysis was limited to a small subset of women presenting in the first trimester, potentially obscuring significant associations, but baseline characteristics of women who attended in the first trimester were not significantly different to women who presented later. No validated questionnaire on diet and exercise in pregnancy currently exists [63], and women’s self-report of exercise (often translated to ‘playing sport’ in Burmese and Karen) and activity in this study was subjective. These limitations were accepted at the study outset, as the primary aim was to develop a baseline understanding of perceptions of marginalised women toward nutritional status in pregnancy.

## Conclusions

There is limited awareness about healthy diets and lifestyle in these marginalised, migrant communities along the Myanmar–Thailand border. This study suggests that simple, culturally appropriate messaging should be provided to women and communities with low health literacy to generate awareness about healthy lifestyles and their effects on pregnancy outcomes as an important element of a broader strategy to address maternal nutrition in this population. However, more studies to determine the effectiveness of a broad range of interventions in LMIC are needed, especially in marginalised migrant populations.

## Paper context

Under- and over-nutrition during pregnancy risk adverse pregnancy and infant outcomes. Understanding perceptions around nutrition in pregnancy can create culturally appropriate interventions for improved health outcomes. This paper describes local perceptions toward nutrition during pregnancy in marginalised populations along the Myanmar–Thailand border. Simple, culturally appropriate messaging provided to women and communities with low health literacy can generate awareness about healthy lifestyles and their importance during pregnancy as part of a broader strategy to address maternal nutrition.
